# An autophagy-related long non-coding RNA signature in tongue squamous cell carcinoma

**DOI:** 10.1186/s12903-023-02806-5

**Published:** 2023-02-22

**Authors:** Yinting Ren, Junlong Da, Junyu Ren, Ye Song, Jingying Han

**Affiliations:** 1 Department of Orthodontics, The Second Affiliated Hospital of Harbin Medical University, Harbin, 150001 Heilongjiang China; 2 Institute of Hard Tissue Development and Regeneration, The Second Affiliated Hospital of Harbin Medical University, Harbin, Heilongjiang China; 3 Oral Implant Center, The Second Affiliated Hospital of Harbin Medical University, Harbin, Heilongjiang China

**Keywords:** Autophagy, Long non-coding RNA, Tongue squamous cell carcinoma, TCGA, Prognostic signature

## Abstract

**Background:**

Tongue squamous cell carcinoma (TSCC) is the most common oral cancer with a poor prognosis. At present, there is not any systematic study on autophagy-related long non-coding RNA (lncRNA) to predict the survival of patients with TSCC.

**Material and methods:**

In this research, the cohort of TSCC patients were obtained from The Cancer Genome Atlas (TCGA) database. Univariate and multivariate Cox regression analysis showed that ten lncRNAs related to autophagy AC010326.3, AL160006.1, AL122010.1, AC139530.1, AC092747.4, AL139287.1, MIR503HG, AC009318.2, LINC01711, and LINC02560 are significantly correlated with prognosis. Based on these lncRNAs, a prognostic signature was established. This signature has an AUC value of 0.782, which accurately distinguishes patients of TSCC into high-risk and low-risk groups in different clinical hierarchical information (such as gender, age, etc.).

**Results:**

The clinical nomogram with autophagy-related lncRNA prognostic characteristics has a concordance index of 0.81, and accurately predicts the survival time at 1-year and 3-year of TSCC patients. Related functional enrichment results indicate that the pathways of the high-risk group are enriched on cancer and autophagy.

**Conclusions:**

The autophagy-related lncRNA prognostic signature established in this study could accurately predict the prognosis of TSCC patients and may be a molecular biomarker and therapeutic target.

**Supplementary Information:**

The online version contains supplementary material available at 10.1186/s12903-023-02806-5.

## Introduction

Tongue squamous cell carcinoma (TSCC) is the most common oral cancer, accounting for 22–49% of all oral cancers [1]. Tongue squamous cell carcinoma has a high degree of malignancy and rate of neck cervical metastasis with a relatively poor prognosis [2, 3]. Nowadays, while surgery-based comprehensive treatment methods are generally adopted, surgical treatment programs usually cause defects of the tongue, which will seriously affect the quality of life in patients [4]. The clinical manifestations of early TSCC are easily ignored by patients, which makes patients lose the best time for treatment [5]. At present, it has been reported that the 5-year survival rate of TSCC is about 56.3% [6], and the current clinical staging needs to be further improved to accurately predict the prognosis of patients.

Autophagy is a highly conserved catabolic process, which is widely distributed in eukaryotes [7]. Under normal physiological conditions, autophagy eliminates dysfunctional organelles and misfolded proteins in the maintenance of cell homeostasis. Dysregulation for autophagy is associated with numerous diseases, such as cancer, metabolic diseases, pathogen infections, and neurodegenerative diseases [8]. Increasing evidence demonstrates that autophagy has a dual role in cancer: autophagy promotes or suppresses the growth of cancer cells by regulating the effects of tumor drugs on cancer cells [9, 10]. The key goal of discovering specific biomarkers of TSCC is to improve the quality of life for patients by early diagnosis and timely treatment. Three proteins, solute carrier family 3 member 2, S100 calcium‐binding protein A2, and IL‐1 receptor antagonist protein were expected to be used as biomarkers for early detection of OSCC. Recent evidence suggests that autophagy modulators may be a potential treatment for the biomarkers (such as protein, RNA, autoantibody and combined biomarkers) in TSCC [11]. Therefore, autophagy-related biomarkers may play an essential role in the early diagnosis and prognosis prediction of TSCC.

Long non-coding RNA (lncRNA), which does not have the ability to encode proteins, plays an indispensable role in all levels of gene function and regulation. LncRNA has plenty of biological functions, for instance, cell proliferation, differentiation, metabolism of RNA, and regulation of epigenetics [12]. Previous studies suggested that lncRNA mediates the expression of autophagy-related genes to coordinate signal pathways with autophagy [13, 14]. Recent studies showed that overexpressed lncRNA CASC9 contributes to the progression of oral squamous cell carcinoma (OSCC) through autophagy-mediated apoptosis [15].

Therefore, we utilized The Cancer Genome Atlas (TCGA) to comprehensively estimate the relationship between the autophagy-related lncRNA and clinicopathological characteristics of tongue squamous cell carcinoma. Because of autophagy-related lncRNA, a prognostic marker was established and its ability to independently predict the prognosis of tongue squamous cell carcinoma patients was evaluated.

## Materials and method

### Data acquisition of tongue squamous cell carcinoma patients

We obtained the RNA-sequencing data sets in HTSeq FPKM format including 15 normal samples and 147 tumor samples from the TCGA database (https://portal.gdc.cancer.gov/). Considering the lack of certain clinical traits, such as 7th edition AJCC stage, TMN staging, etc., the samples of 134 patients with complete clinical information are retained. Since the data of this study are obtained from public database, the approval of the ethics committee is not necessary. The data of all patients are provided in the Additional files 1, 2, 3, 4, 5 and 6.

### Screening of autophagy-related lncRNAs

The 232 autophagy-associated genes are collected through the Human Autophagy Database (HADb, http://www.autophagy.lu/clustering/index.html), which is a public database containing human genetic information related to autophagy that has been described so far [16]. All the lncRNA was isolated and recognized from RNA-sequencing data sets by Perl programming language. Pearson correlation coefficient is served to describe the pertinence between the expression of autophagy-related genes and lncRNAs by R. These are the criteria used to shortlist autophagy-related lncRNAs: *p* < 0.05 and |*R*^2^| > 0.3 [17].

### Construction of prognostic autophagy-related lncRNAs signature

We identified autophagy-related lncRNAs via a univariate Cox regression model by R. The lncRNAs that were significant (*p* < 0.05) with overall survival of TSCC patients were chosen. The multivariate Cox stepwise regression model was performed to evaluate the potential of autophagy-related lncRNAs as independent prognostic factors. Based on the minimum Akaike information criterion (AIC) value, we selected the first-rank autophagy-related lncRNA to construct a risk score for each patient with TSCC. The risk score formula of autophagy-related lncRNAs prognosis signature was shown as the following: Risk score = ${\sum }_{\theta =1}^{n}Coef(\theta )\times Expr(\theta )$. The *n* represents the number of lncRNAs used to construct the model. $Coef(\theta )$ represents the coefficient in the regression model, and $Expr(\theta )$ is defined as the expression of each lncRNA in this formula [17]. 146 patients of TCGA-TSCC were divided into two groups with low-risk or high-risk score according to the median value of risk score.

### Co-expression network of mRNA and autophagy-related lncRNAs

The construction of lncRNAs and mRNA co-expression network on TSCC was to gain insight into the correlation between autophagy-related lncRNAs and co-expression of autophagy-related mRNA. Pearson’s coefficients > 0.3 was used to shortlist mRNA that was related to lncRNA. We applied Cytoscape software (version 3.7.1) to showcase the relationship between mRNA and autophagy-related lncRNA for visualization.

### Construction and verification of nomogram

We established a nomogram to predict the 1-year and 3-year survival rates of TSCC by integrating the corresponding clinical information (including age, gender, grade, AJCC stage, T, and N stage) and risk score. Subsequently, we utilized the concordance index (C-index) to appraise the reliability of this nomogram. The C-index refers to the consistency between the actual probability of the outcome and the predicted probability. Generally, the closer the value of the C-index is to 1, the higher the accuracy of the predictive ability of the nomogram. Both the nomogram and calibration curves are based on the RMS package [18] in the R software (version 4.0.3).

### Gene set enrichment analysis

To explore the differences in biological annotations and pathways between the high-risk and low-risk groups, we applied the gene set enrichment analysis (GSEA) software (version 4.1.0, https://www.gsea-msigdb.org) to obtain the results [19]. The Molecular Signatures Database (MSigDB) of c2 (c2.cp.kegg.v7.2.symbols.gmt) was selected as the reference gene sets database, and the number of permutations was 1000. False discovery rate (FDR) < 0.25 and *p* < 0.05 were considered significant.

### Statistical analysis and data availability

The Cox regression analysis was performed to estimate the hazard ratio (HR) and 95% confidence interval (CI) values using R software (version 4.0.3). The correlation of the autophagy-related lncRNAs was calculated by Pearson correlation. *p* < 0.05 were regarded as statistically significant. All data and code can be available in Additional file 1, 2, 3, 4, 5, 6 and 7 related to this study.

## Results

### Identification of autophagy-related lncRNAs with prognostic significance in TSCC patients of TCGA database

We identified 14,142 lncRNAs by analyzing the RNA-seq of 162 TSCC patients samples obtained from TCGA. A total of 232 autophagy-related genes were downloaded from the HADb database and we acquired the corresponding expression levels of the patient samples. By setting the threshold of Pearson coefficient and *p* value, 941 lncRNAs related to autophagy were shortlisted out. The univariate Cox regression analysis was used to obtain 25 autophagy-related lncRNAs that were significantly related to the overall survival time of TSCC patients. Based on the minimum AIC (Table 1), multivariate Cox analysis revealed that ten lncRNAs AC010326.3, AL160006.1, AL122010.1, AC139530.1, AC092747.4, AL139287.1, MIR503HG, AC009318.2, LINC01711, and LINC02560 were the best candidates for constructing a prognostic model (Table 2). Among the ten autophagy-related lncRNAs, risk factors of AC010326.3 and AL139287.1 with HR values were greater than 1, while the rest were considered as protective factors with HR values that were less than 1.

**Table 1:** AIC for the models

Model	Prognostic signature combination	AIC
1	AC010326.3 + AL160006.1 + AL122010.1 + AL136295.7 + AC139530.1 + AC092747.4 + AL139287.1 + MIR503HG + AC009318.2 + ‘RTCA-AS1’ + LINC01711 + LINC02560	467.32
2	AC010326.3 + AL160006.1 + AL122010.1 + AC139530.1 + AC092747.4 + AL139287.1 + MIR503HG + AC009318.2 + RTCA-AS1 + LINC01711 + LINC02560	466.96
3	AC010326.3 + AL160006.1 + AL122010.1 + AC139530.1 + AC092747.4 + AL139287.1 + MIR503HG + AC009318.2 + LINC01711 + LINC02560	466.64

**Table 2:** Multivariate Cox analysis results based on autophagy-related lncRNA

LncRNA	Coefficient	Hazard ratio	95% Confidence interval	Prognostic value
AC010326.3	0.3919	1.4798	0.9768–2.2417	Risk
AL160006.1	− 0.3971	0.6723	0.4529–0.9978	Protective
AL122010.1	− 0.4357	0.6468	0.3555–1.1767	Protective
AC139530.1	− 0.9133	0.4012	0.1923–0.8372	Protective
AC092747.4	− 0.1894	0.8274	0.6770–1.0112	Protective
AL139287.1	0.2274	1.2553	1.0076–1.5640	Risk
MIR503HG	− 0.2733	0.7609	0.5699–1.0159	Protective
AC009318.2	− 0.7450	0.4747	0.2130–1.0580	Protective
LINC01711	− 1.3486	0.2596	0.1021–0.6600	Protective
LINC02560	− 0.0652	0.9369	0.8897–0.9866	Protective

### Prognostic evaluation of autophagy-related lncRNAs in TSCC

**Fig. 1:**
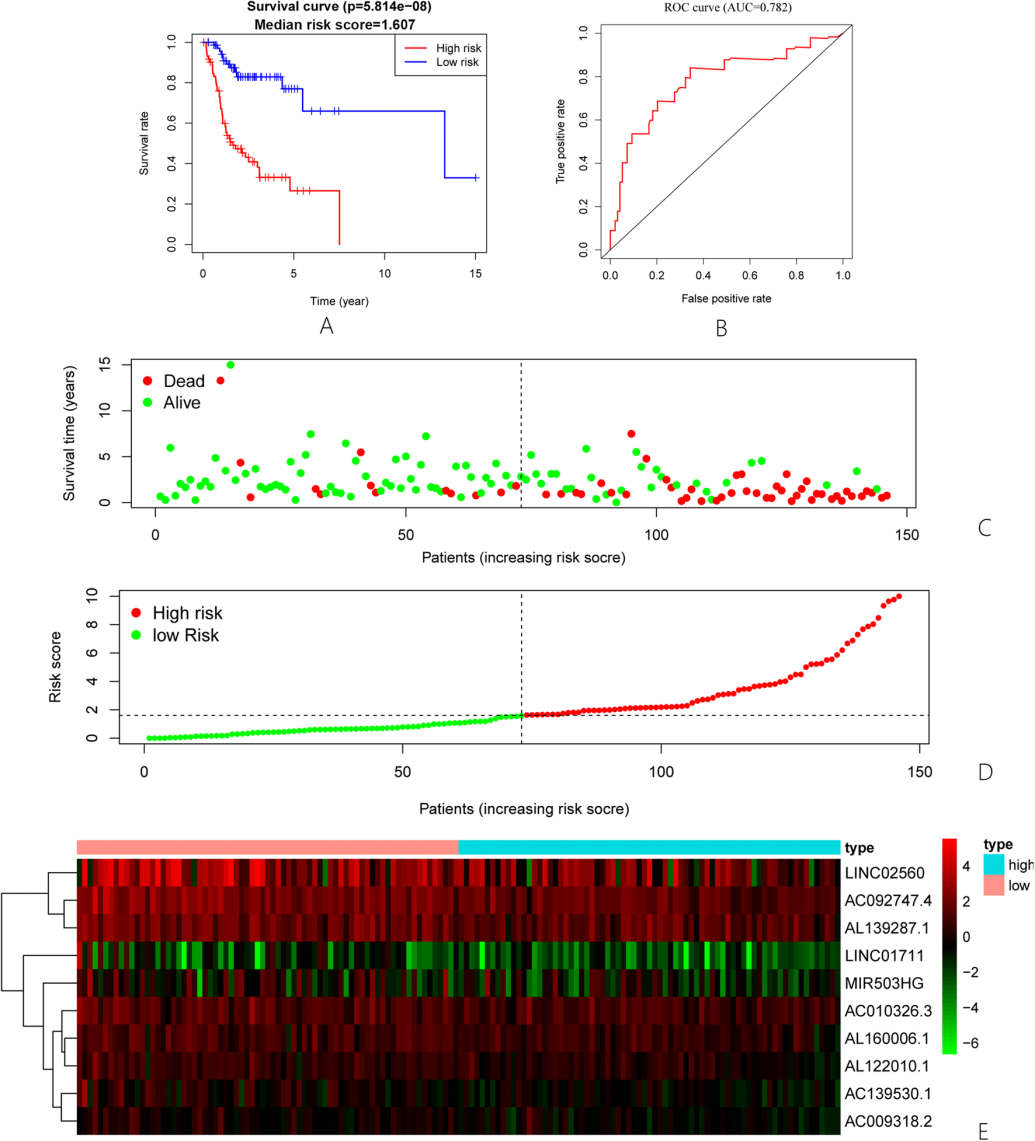
Verification of prognostic signatures of lncRNAs associated with autophagy in TSCC patients. **A** The overall survival time of TSCC patients in the high-risk and low-risk groups was analyzed by the median risk score. The survival curve shows that there were significant statistical differences between high-risk and low-risk survival rates. **B** ROC curve displayed the accuracy of the autophagy-related lncRNA prognostic prognosis model (AUC = 0.782) in predicting survival times of TSCC patients from the TCGA database. **C** The relationship between survival status and risk score of TSCC patients based on prognosis signature. **D** The distribution of high-risk and low-risk scores in TSCC patients. The risk score increased from green to red. **E** Thermography showed the expression levels of ten autophagy-related lncRNAs in patients with high-risk and low-risk

To investigate whether autophagy-related lncRNAs are correlated to the prognosis of TSCC, we have established a risk scoring model. The model is a summation of the expression of lncRNA multiplied by the corresponding coefficient, namely risk score = (0.3919 × expression of AC010326.3) + (− 0.3971 × expression of AL160006.1) + (− 0.4357 × expression of AL122010.1) + (− 0.9133 × expression of AC139530.1) + (− 0.1894 × expression of AC092747.4) + (0.2274 × expression of AL139287.1) + (− 0.2733 × expression of MIR503HG) + (− 0.7450 × expression of AC009318.2) + (− 1.3486 × expression of LINC01711) + (− 0.0652 × expression of LINC02560). TSCC patients were divided into high-risk and low-risk groups based on the median risk score of 1.607. In order to verify the significance of the high-risk and low-risk groups in the OS of TSCC patients, Kaplan–Meier survival curve analysis was used to create the risk survival curve. As the survival time increases, the survival rate of the high-risk group drops sharply, while the result of the low-risk group is better than the high-risk group (Fig. 1A). The areas under the time-dependent receiver operating characteristic (ROC) curve of the autophagy-related lncRNAs prognostic model were 0.782 (Fig. 1B), which suggests the prognosis signature has the potential in predicting survival. Figure 1C shows the survival status between different groups, and Fig. 1D depicts the classification of TSCC patients into high-risk and low-risk groups based on risk scores. These results indicate that the prognosis model based on autophagy-related lncRNAs could accurately classify the survival status and the risk of TSCC patients. The heat map displays the expression levels of ten autophagy-related lncRNAs in the high-risk and low-risk groups (Fig. 1E). The color changes from red to green, indicating a downward trend from high to low expression levels.

### The correlation between the prognostic model constructed by autophagy-related lncRNA and clinical characteristics 

**Fig. 2:**
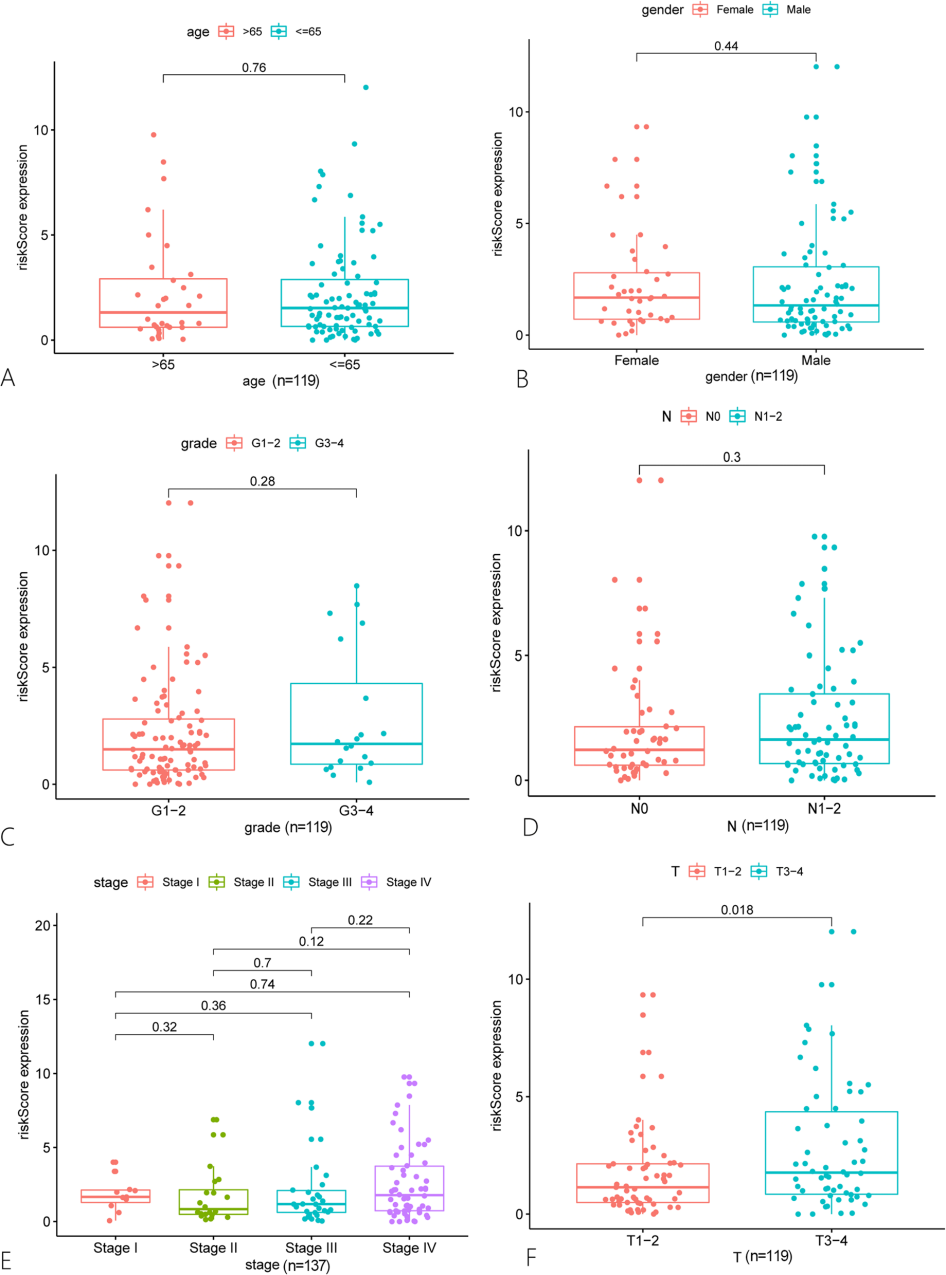
The correlation between different clinicopathological features and the risk score of autophagy-related lncRNAs. **A–F** show respectively the difference of risk score expression in age (≥ 65 years; < 65 years), gender (male; female), tumor grade (G1/G2; G3/G4), N stage (N0; N1–N2), AJCC stage, and T stage (S1/S2; S3/4). And the T stage is significantly related to risk score. The statistical analyses of **A**–**D** and **F** were performed by Wilcoxon, while **E** was performed by Anova

We gained the clinical data of TSCC patients from the TCGA database and analyzed the correlation between the risk score and clinicopathological characteristics. The results show that there is no significant statistical difference between the risk score and the risk score and age, gender, grade, AJCC stage, and N stage of TSCC patients (Fig. 2A–E). Meanwhile, in the T stage, patients with T3–T4 had a higher risk score compared with T1–T2 (Fig. 2F, *p* < 0.05). These analysis results imply that the autophagy-related lncRNA risk score is related to the T stage of TSCC patients.

**Fig. 3.**
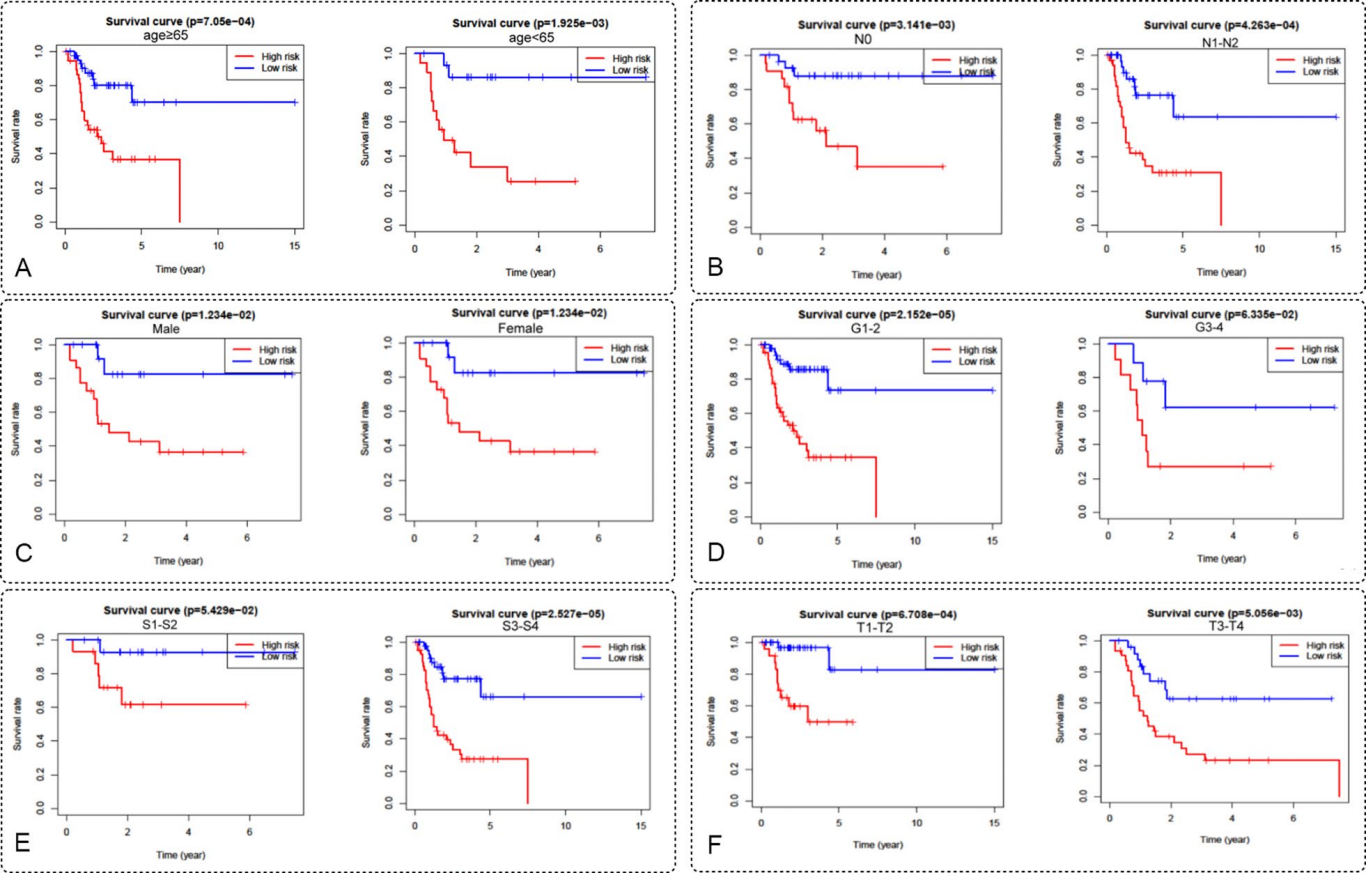
The survival rates of high- and low-risk TSCC patients stratified by different clinicopathological characteristics. Kaplan Meier survival curve analysis shows overall survival (OS) rates of high- and low-risk BCLA patients from the TCGA database stratified by **A** age (≥ 65 years; < 65 years), **B** N (N0; N1–N2), **C** gender (male; female), **D** tumor grade (G1/G2; G3/G4), **E** AJCC stage (S1/S2; S3/4), **F** T (T1/T2; T3/T4).

We further stratified the clinical information of TSCC patients to estimate the autophagy-related lncRNA prognostic model. As shown in Fig. 3, we draw survival curves of this prognostic models based on age (age ≥ 65: *p* = 7.05e−04; age < 65: *p* = 1.925e−03), gender (male: *p* = 1.234e−02; female: *p* = 1.234e−02), tumor grade (G1–2: *p* = 2.152e−05; G3–4: *p* = 6.335e−02), AJCC stage (Stage I–II: *p* = 5.429e−02; Stage III–IV: *p* = 2.527e−05), T stage (T1–2: *p* = 6.708e−04; T3–4: *p* = 5.056e−03) and N stage (N0: *p* = 3.141e−03; N1–2: *p* = 4.263e−04), respectively. Those suggest that the patients with low-risk scores have significantly longer OS times than high-risk scores.

### Autophagy-related lncRNA prognostic model is an independent prognostic factor for TSCC

**Fig. 4:**
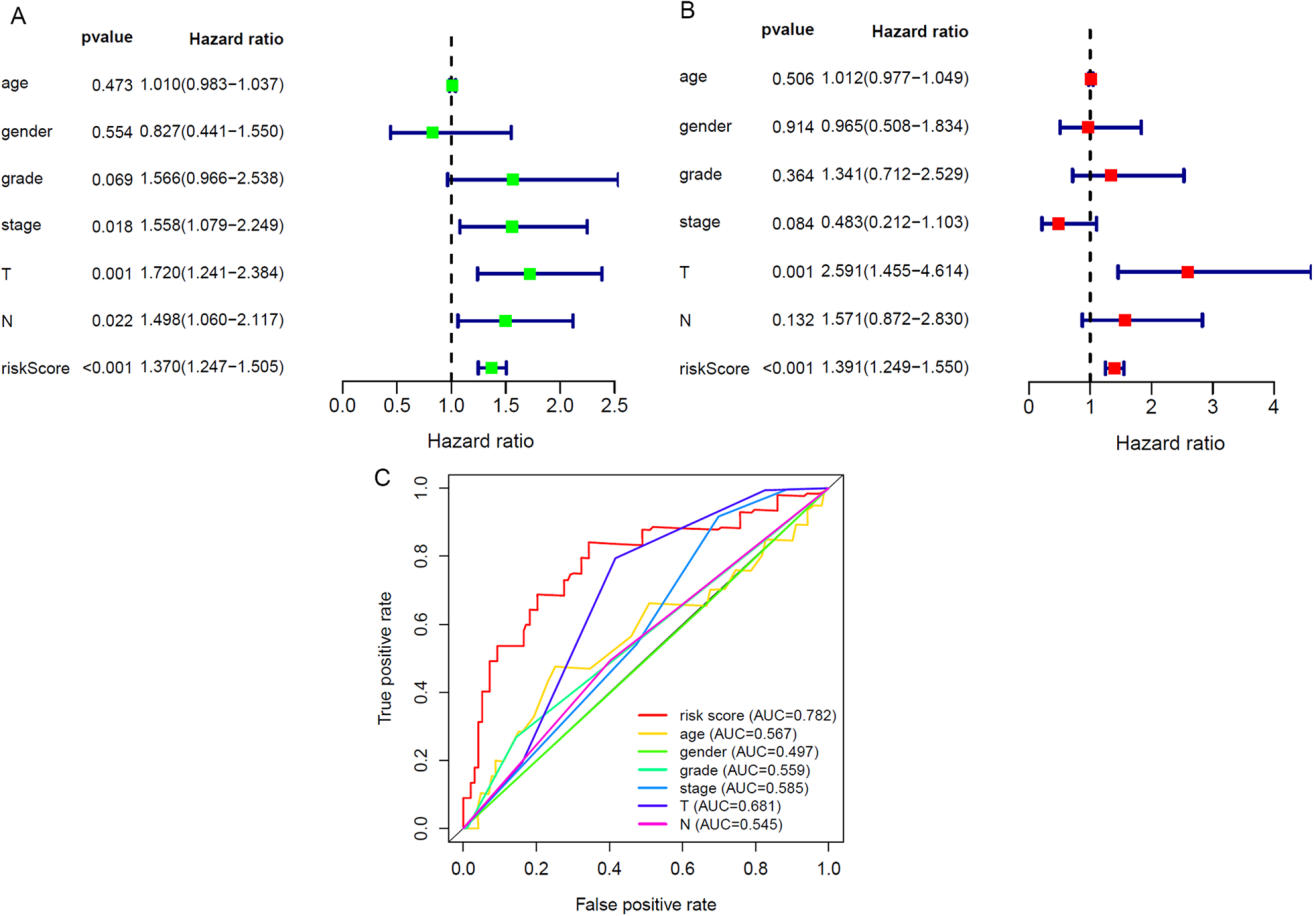
Evaluating the accuracy of autophagy related lncRNAs prognostic signatures risk score and clinicopathological features in TSCC. Prognostic indicators based on autophagy-related lncRNAs showed great predictive performance. **A** Univariate Cox regression analysis showed the relationship between OS and autophagy related lncRNAs prognostic signature risk score and other clinical characteristics. **B** Multivariate Cox regression exhibited that risk score and T stage were independent prognostic factors of TSCC (*p* < 0.01). **C** ROC curves demonstrated a comparison of accuracy of predicting prognosis risk score between autophagy related lncRNAs prognostic signatures and other clinicopathological features

By way of determining whether the prognostic signature we constructed could be an independent prognostic factor for TSCC, we applied univariate and multivariate Cox regression analysis. The results of univariate Cox regression analysis indicated that the Hazard Ratio of AJCC stage (*p* = 0.018), T stage (*p* = 0.001), N stage (*p* = 0.022), and risk score (*p* < 0.001) in TSCC patients were greater than 1. It shows that these factors are significantly related to the OS of patients (Fig. 4A). In the results of multivariate Cox regression analysis, T stage (*p* = 0.001) and risk score (*p* < 0.001) were significantly correlated with OS (Fig. 4B). We further plotted the AUC curve, as shown in Fig. 4C, the risk score of the autophagy-related lncRNA prognostic signature has an AUC of 0.782, which is higher than age (AUC = 0.567), gender (AUC = 0.497), grade (AUC = 0.559), stage (AUC = 0.585), T stage (AUC = 0.681), and N stage (AUC = 0.545). These data suggest that the autophagy-related lncRNA prognostic signature is an independent prognostic factor for TSCC patients.

### Evaluation of nomogram composed of autophagy-related lncRNA prognostic signatures

**Fig. 5:**
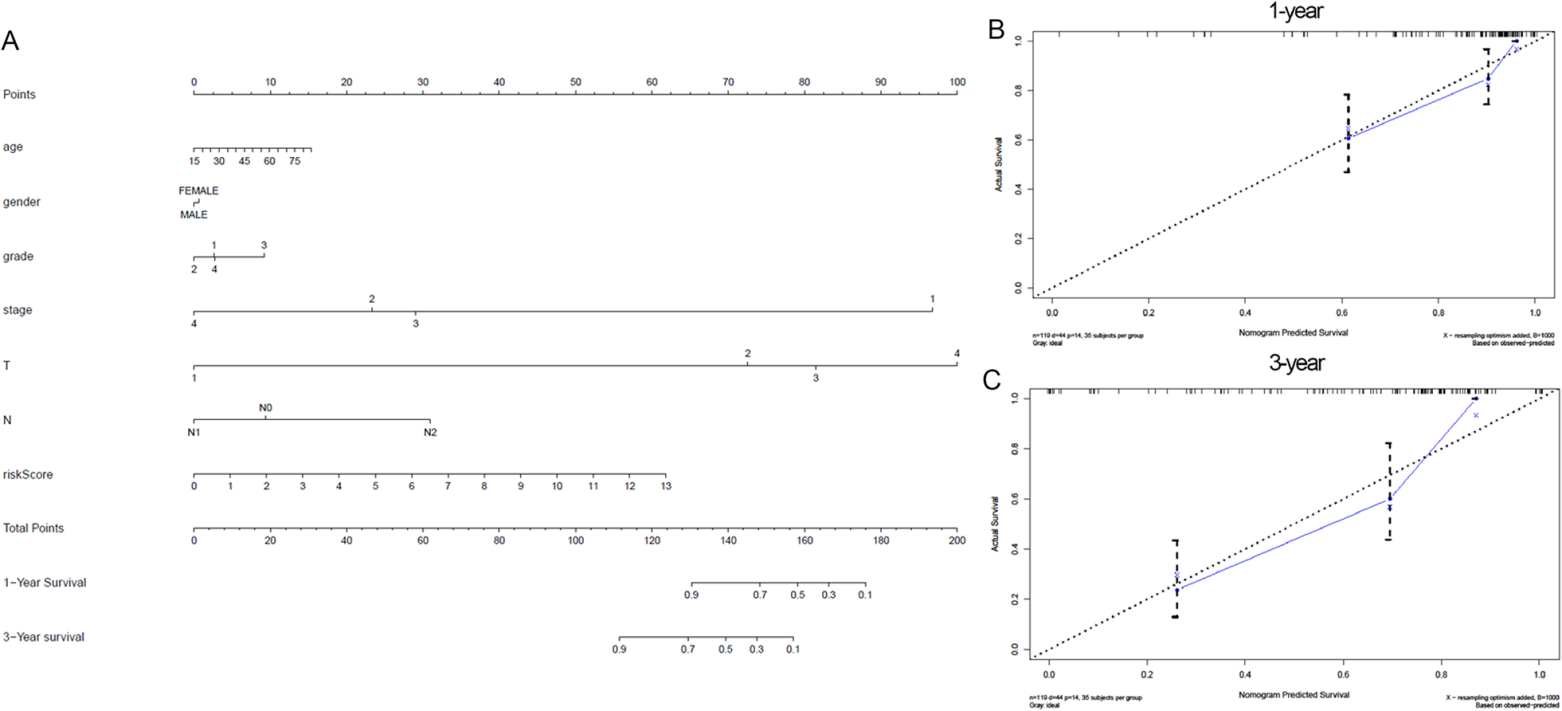
The evaluation of prognostic models based on ten autophagy-related lncRNAs. Construction and validation of the prognostic nomogram with autophagy-related lncRNA prognostic signature risk score as one of the parameters. **A** The establishment of nomograms containing autophagy-related lncRNA prognostic signatures risk score and clinical features. **B** and **C** are calibration plots of 1-year and 3-year predicted survival rates and actual survival rates respectively, which evaluate the veracity of prognostic models

We constructed a nomogram with a concordance index (C-index) value of 0.81 composed of clinicopathological characteristics, including age, gender, grade, AJCC stage, T stage, N stage, and risk score of prognostic signatures (Fig. 5A). Figure 5B and C show the results of the 1-year and 3-year calibration curve analysis respectively. As shown in the figures, compared with the reference line, the actual survival time is the same as the predicted survival time. This result suggests that the autophagy-related lncRNA prognostic signature that we constructed can accurately predict the prognostic survival time of TSCC patients.

### Co-expression network construction and related functional enrichment analysis

**Fig. 6:**
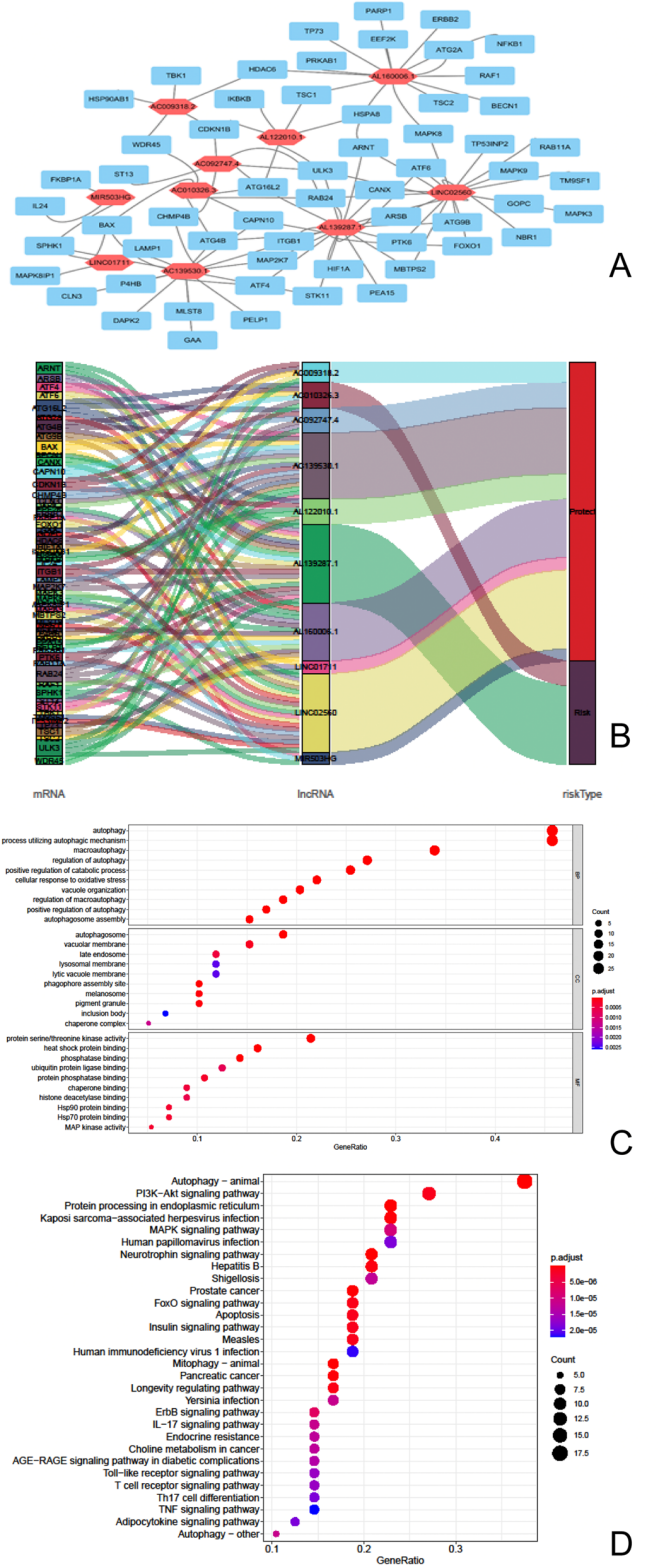
Construction and functional enrichment analysis of autophagy-related lncRNA-mRNA co-expression network. **A** Co-expression network of lncRNA and mRNA related to prognosis. The red hexagon represents autophagy-related lncRNA, and the blue box represents autophagy-related genes. Each line represents its corresponding relationship. The visualization was created by Cytoscape version 3.7.1. **B** Sankey diagram shows the relationship between autophagy-related mRNA, lncRNA, and risk types. **C** GO analysis results show that the co-expression network is enriched in biological processes (BP), cellular components (CC) and molecular functions (MF). **D** KEGG pathway analysis results display that there are abundant signal pathways including Autophagy, Pl3k-Akt signaling pathway, Protein processing in endoplasmic reticulum, MAPK signaling pathway and so on related to co-expressed expression network

In order to further clarify the relationship between autophagy-related mRNA in the TSCC patients and the ten autophagy-related lncRNAs selected, we obtained 97 autophagy-related mRNAs through the threshold of the Pearson correlation coefficient (|R^2^| > 0.3) to construct the mRNA-lncRNA co-expression network via Cytoscape (version 3.7.1) (Fig. 6A). Then, the sankey diagram (Fig. 6B) shows the correspondence between autophagy-related lncRNAs and risk factors (risk or protective factors) in the co-expression network. Figure 6C depicted the top ten enriched terms for biological processes (BP), cellular components (CC), and molecular functions (MF) in gene ontology (GO) enrichment analysis. The top three terms in BP are autophagy, process utilizing autophagic mechanism, and macroautophagy. Autophagosome, vacuum membrane, and late endosome are the top three terms in CC. Protein serine/threonine kinase activity, heat shock protein binding, and phosphatase binding are the top three terms in CC. In the KEGG pathway analysis, we showed the top thirty enriched pathways. As shown in Fig. 6D, autophagy, PI3K-Akt signaling pathway, protein processing in endoplasmic reticulum pathway played an indispensable role in the co-expression of mRNA and autophagy-related lncRNAs.

### Gene set enrichment analysis

**Fig. 7:**
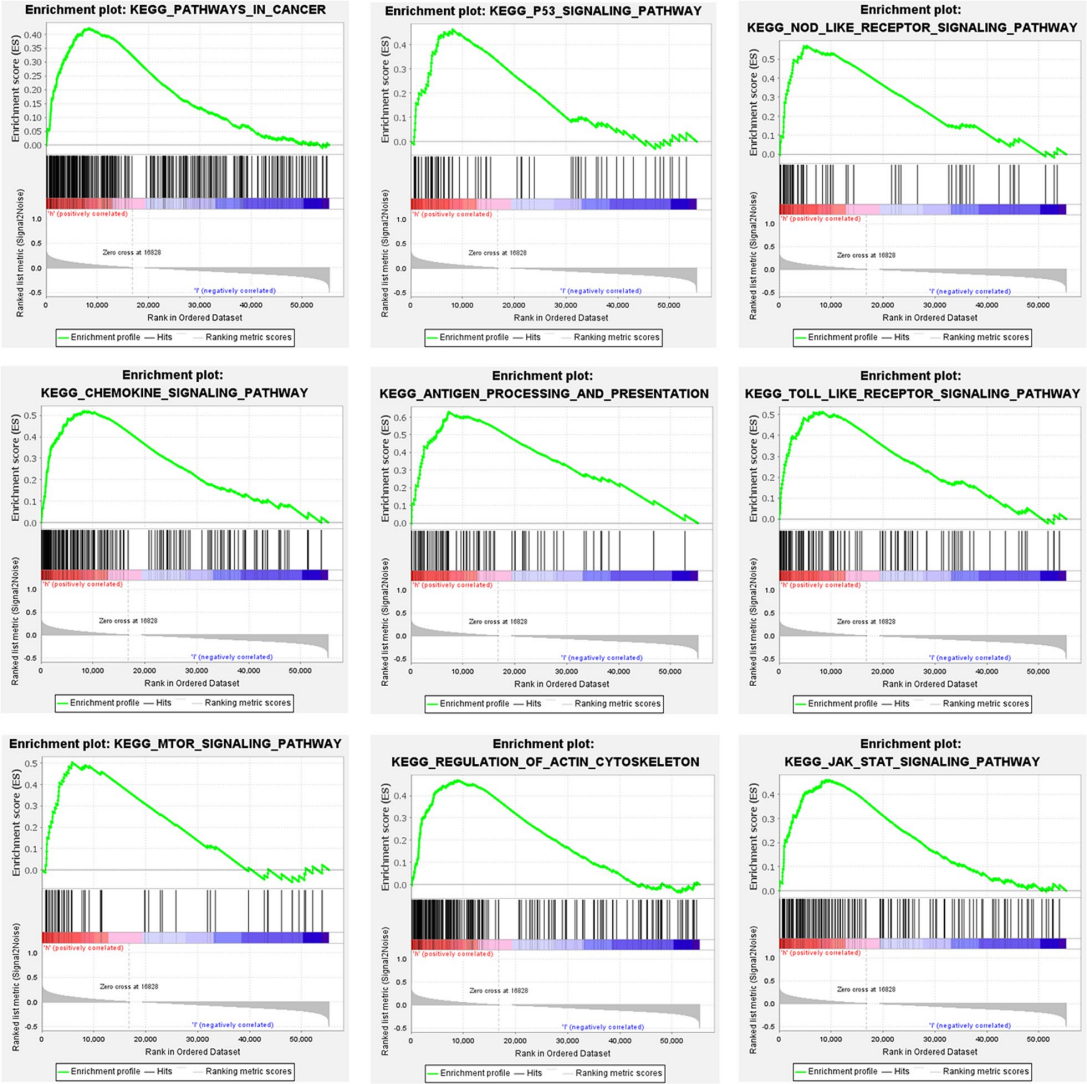
Autophagy-related lncRNA prognosis signature risk score GSEA results of high-risk TSCC patients. Gene set enrichment analysis indicated significant enrichment of hallmark cancer-related and autophagy-related pathways in the high-risk group based on TCGA dataset

Further functional GSEA revealed that the altered genes (including mRNA and lncRNA) were significantly enriched in autophagy, cancer, and immune-related pathways, for example, pathways in cancer (*p* = 0.032), mTOR signaling pathway (*p* = 0.008), p53 signaling pathway (*p* = 0.045), NOD-like receptor signaling pathway (*p* = 0.006), Toll-like receptor signaling pathway (*p* = 0.021), regulation of actin cytoskeleton (*p* = 0.01), chemokine signaling pathway (*p* = 0.019) in patients with high risk of TSCC (Fig. 7). This indicates that changes in these pathways may affect the prognosis of TSCC patients. These results provide new insights into the pathogenesis of TSCC and cancer-targeted treatment strategies (such as immunotherapy).

## Discussion

OSCC is the most common and main malignant tumor in head and neck cancer [20]. The International Union Against Cancer has included OSCC as one of the most common malignant cancers, with approximately 275,000 cases worldwide each year [21]. Oral TSCC is the main component of OSCC. Compared with other malignant tumors of the head and neck, tongue cancer has strong invasiveness, high metastasis and, high recurrence, which obviously affect the functions of chewing, swallowing, and breathing [22]. Autophagy has been confirmed to be associated with the development of various cancers, including TSCC [23]. Therefore, biomarkers related to autophagy may play an important role in the early diagnosis and targeted therapy of tongue cancer. Previous studies focused on the regulation of genes related to autophagy in TSCC [24, 25].

More and more reports indicate that lncRNA, as a non-coding molecule, is closely related to autophagy in the malignant progression of some cancers [26]. Therefore, lncRNA is a potential biomarker to predict the prognosis of tumor patients. As far as we know, there is no systematic method to identify the lncRNA signature used to predict the survival of TSCC patients. Therefore, this study established autophagy-related lncRNA signatures to accurately predict the prognosis of TSCC.

In this study, we first shortlisted lncRNAs related to autophagy in samples of TSCC patients from the TCGA database and identified 25 lncRNAs related to survival time through univariate Cox regression. The ten lncRNAs related to autophagy were identified via multivariate Cox regression analysis. In addition, based on the median risk score calculated by the expression level of lncRNA, the patients with TSCC were divided into high-risk and low-risk groups. There were significant statistical differences between high-risk and low-risk survival rates (*p* = 5.814e−08). The ROC curve confirms the accuracy of autophagy-related lncRNA prognostic signatures in TSCC patients (AUC = 0.782). We can conclude from univariate and multivariate Cox regression analysis that autophagy-related lncRNA prognostic signature is an independent prognostic factor significantly related to OS. In different clinical categories (such as gender, age, grade, AJCC stage, T stage, and N stage), autophagy-related lncRNA prognostic signatures can accurately predict the survival outcome of the different groups, indicating that this prognostic model is accurate and reliable.

Compared with traditional clinical features, autophagy-related lncRNA prognostic models are more accurate predictors. For many cancers, the nomogram is superior to the traditional TNM staging system because of its applicability and accuracy, so it has been proposed as an alternative or as a new standard [27]. Therefore, based on current clinical information and autophagy-related lncRNA prognostic signatures, we developed a nomogram for TSCC. The calibration curve analysis shows that the actual 1-year and 3-year survival times are similar to the actual values of TSCC. To summarise, the autophagy-related lncRNA prognostic signatures we established have great potential for clinical application.

Autophagy is a highly conservative biological process to maintain cell metabolism. Under pathological or physiological conditions, autophagosomes captures and degrades intracellular components, such as proteins and organelles in lysosomes [28]. The exact mechanism of autophagy in cancer has not yet been fully elucidated and the dual effects of autophagy on the inhibition and promotion of various tumors remain controversial [29]. AMPK and mTOR negatively regulate tumor suppressor factors, leading to the induction of autophagy and tumor suppression in the early stage of cancer [30]. On the other hand, oncogenes may be activated by class I PI3K, AKT, and mTOR, contributing to the inhibition of autophagy and the promotion of cancer [31]. In recent years, there have been more studies about lncRNA that regulate autophagy to influence the biological behavior of OSCC [32–36]. LncRNA HOTAIR accelerates the proliferation, migration, and invasion of OSCC cells by raising the expression of microtubule-associated protein 1 light chain 3B (MAP1LC3B), Beclin 1 (BECN1), autophagy-related gene (ATG3 and ATG7) [37]. Yang et al. reported that lncRNA CASC9 was significantly up-regulated in OSCC. The knockout of CASC9 significantly reduced the expression level of p-mTOR, BCL-2, p-AKT, and P62, which demonstrated that lncRNA CASC9 suppresses autophagy-mediated apoptosis by AKT/mTOR pathway to promote OSCC progression [15]. Therefore, we identified ten lncRNAs related to autophagy and constructed a lncRNA-mRNA co-expression network to evaluate their functions. The terms and pathways related to autophagy have been significantly enriched (*p* < 0.05) in the functional analysis of GO, KEGG, and GSEA. It implies that autophagy plays a pivotal role in the progression of TSCC and might have a potential as a therapeutic target, which is consistent with previous studies [38].

Our study has several limitations. First, the sample size of TSCC patients we obtained in the TCGA database is limited, which may affect the accuracy of the prognostic model we constructed. Second, the sample information based on the 7th edition AJCC classification is limited since the 8th edition AJCC has a better prognostic value than the 7th edition. Finally, our findings need to be further validated in other independent cohorts to determine the robustness of autophagy-related lncRNA prognostic signature.

In summary, the constructed autophagy-related lncRNA prognostic signature can accurately predict the prognosis of TSCC patients. A nomogram containing lncRNAs and other clinicopathological characteristics is established, and our research suggests that the nomogram could correctly predict the survival outcomes of TSCC patients. These ten lncRNAs are potential prognostic biomarkers and possible targets for TSCC treatment.

## Supplementary Information

**Additional file 1.** Gene-symbol.

**Additional file 2.** lncRNA.

**Additional file 3.** Autophagy-related-gene.

**Additional file 4.** Autophagy-related-gene-expression.

**Additional file 5.** Autophagy-related-lncRNA-expression.

**Additional file 6.** Clinical information.

**Additional file 7.** Code.

## Data Availability

The datasets for this study can be found in the TCGA (https://portal.gdc.cancer.gov/). Code and related files, including gene-symbol, lncRNA, Autophagy-related-gene, Autophagy-related-gene-expression, Autophagy-related-lncRNA-expression, Clinical information, and Code have been uploaded to the supplemental material.
