# Supplementary material for: An autophagy-related long non-coding RNA signature in tongue squamous cell carcinoma
Source: BMC Oral Health. 2023 Feb 22;23:120. doi: 10.1186/s12903-023-02806-5 (PMC9945660; doi:10.1186/s12903-023-02806-5)
Supplement: Supplementary file 7 — Additional file 7. Code. [file 12903_2023_2806_MOESM7_ESM.docx]

**This text is all the R code used in this manuscript. ## represents the subheadings of each section.**

##Screening the expression of autophagy related genes.

if (!requireNamespace("BiocManager", quietly = TRUE))

install.packages("BiocManager")

BiocManager::install("limma")

library(limma)

setwd("D:\\autophagy-related")

rt=read.table("protein.txt",sep="\t",header=T,check.names=F)

rt=as.matrix(rt)

rownames(rt)=rt[,1]

exp=rt[,2:ncol(rt)]

dimnames=list(rownames(exp),colnames(exp))

data=matrix(as.numeric(as.matrix(exp)),nrow=nrow(exp),dimnames=dimnames)

data=avereps(data)

data=data[rowMeans(data)>0,]

gene=read.table("gene.txt", header=F, check.names=F, sep="\t")

sameGene=intersect(as.vector(gene[,1]),rownames(data))

geneExp=data[sameGene,]

out=rbind(ID=colnames(geneExp),geneExp)

write.table(out,file="ARGexp.txt",sep="\t",quote=F,col.names=F)

##Screening of lncRNAs Associated with Autophagy

if (!requireNamespace("BiocManager", quietly = TRUE))

install.packages("BiocManager")

BiocManager::install("limma")

library(limma)

setwd("D:\\autophagy-related ")

corFilter=0.3

pvalueFilter=0.001

rt = read.table("lncRNA.txt",header=T,sep="\t",check.names=F)

rt=as.matrix(rt)

rownames(rt)=rt[,1]

exp=rt[,2:ncol(rt)]

dimnames=list(rownames(exp),colnames(exp))

lncRNA=matrix(as.numeric(as.matrix(exp)),nrow=nrow(exp),dimnames=dimnames)

lncRNA=avereps(lncRNA)

lncRNA=lncRNA[rowMeans(lncRNA)>0.5,]

group=sapply(strsplit(colnames(lncRNA),"\\-"),"[",4)

group=sapply(strsplit(group,""),"[",1)

group=gsub("2","1",group)

lncRNA=lncRNA[,group==0]

rt = read.table("ARGexp.txt",header=T,sep="\t",check.names=F)

rt=as.matrix(rt)

rownames(rt)=rt[,1]

exp=rt[,2:ncol(rt)]

dimnames=list(rownames(exp),colnames(exp))

ARGgene=matrix(as.numeric(as.matrix(exp)),nrow=nrow(exp),dimnames=dimnames)

ARGgene=avereps(ARGgene)

ARGgene=ARGgene[rowMeans(ARGgene)>0.5,]

group=sapply(strsplit(colnames(ARGgene),"\\-"),"[",4)

group=sapply(strsplit(group,""),"[",1)

group=gsub("2","1",group)

ARGgene=ARGgene[,group==0]

outTab=data.frame()

for(i in row.names(lncRNA)){

if(sd(lncRNA[i,])>0.5){

for(j in row.names(ARGgene)){

x=as.numeric(lncRNA[i,])

y=as.numeric(ARGgene[j,])

corT=cor.test(x,y)

cor=corT$estimate

pvalue=corT$p.value

if((abs(cor)>corFilter) & (pvalue<pvalueFilter)){

outTab=rbind(outTab,cbind(ARGgene=j,lncRNA=i,cor,pvalue))

}

}

}

}

write.table(file="corResult.txt",outTab,sep="\t",quote=F,row.names=F)

ARGlncRNA=unique(as.vector(outTab[,"lncRNA"]))

ARGlncRNAexp=lncRNA[ARGlncRNA,]

ARGlncRNAexp=rbind(ID=colnames(ARGlncRNAexp),ARGlncRNAexp)

write.table(ARGlncRNAexp,file="ARGlncRNAexp.txt",sep="\t",quote=F,col.names=F)

## uniCox

install.packages("survival")

library(survival)

pFilter=0.05

setwd("D:\\autophagy-related ")

rt=read.table("expTime.txt",header=T,sep="\t",check.names=F,row.names=1)

rt$futime=rt$futime/365

outTab=data.frame()

sigGenes=c("futime","fustat")

for(gene in colnames(rt[,3:ncol(rt)])){

if(sd(rt[,gene])<0.01){

next}

a=rt[,gene]<=median(rt[,gene])

diff=survdiff(Surv(futime, fustat) ~a,data = rt)

pValue=1-pchisq(diff$chisq,df=1)

fit=survfit(Surv(futime, fustat) ~ a, data = rt)

cox=coxph(Surv(futime, fustat) ~ rt[,gene], data = rt)

coxSummary = summary(cox)

coxP=coxSummary$coefficients[,"Pr(>|z|)"]

if((pValue<pFilter) & (coxP<pFilter)){

sigGenes=c(sigGenes,gene)

outTab=rbind(outTab,

cbind(gene=gene,

KM=pValue,

B=coxSummary$coefficients[,"coef"],

SE=coxSummary$coefficients[,"se(coef)"],

HR=coxSummary$conf.int[,"exp(coef)"],

HR.95L=coxSummary$conf.int[,"lower .95"],

HR.95H=coxSummary$conf.int[,"upper .95"],

pvalue=coxP) )

}

}

write.table(outTab,file="uniCox.xls",sep="\t",row.names=F,quote=F)

uniSigExp=rt[,sigGenes]

uniSigExp=cbind(id=row.names(uniSigExp),uniSigExp)

write.table(uniSigExp,file="uniSigExp.txt",sep="\t",row.names=F,quote=F)

##multiCox

install.packages('survival')

library(survival)

setwd("D:\\autophagy-related ")

rt=read.table("uniSigExp.txt",header=T,sep="\t",check.names=F,row.names=1)

multiCox=coxph(Surv(futime, fustat) ~ ., data = rt)

multiCox=step(multiCox,direction = "both")

multiCoxSum=summary(multiCox)

outTab=data.frame()

outTab=cbind(

coef=multiCoxSum$coefficients[,"coef"],

HR=multiCoxSum$conf.int[,"exp(coef)"])

outTab=cbind(id=row.names(outTab),outTab)

outTab=gsub("`","",outTab)

write.table(outTab,file="multiCox.txt",sep="\t",row.names=F,quote=F)

riskScore=predict(multiCox,type="risk",newdata=rt)

coxGene=rownames(multiCoxSum$coefficients)

coxGene=gsub("`","",coxGene)

outCol=c("futime","fustat",coxGene)

risk=as.vector(ifelse(riskScore>median(riskScore),"high","low"))

write.table(cbind(id=rownames(cbind(rt[,outCol],riskScore,risk)),cbind(rt[,outCol],riskScore,risk)),

file="risk.txt",

sep="\t",

quote=F,

row.names=F)

##Expression of risk score in clinical information

if (!requireNamespace("BiocManager", quietly = TRUE))

install.packages("BiocManager")

BiocManager::install("limma")

install.packages("ggpubr")

library(limma)

library(ggpubr)

setwd("D:\\autophagy-related ")

file="Riskscore.txt"

rt=read.table(file,sep="\t",header=T,check.names=F,row.names=1)

cli=read.table("N.txt",sep="\t",header=T,check.names=F,row.names=1)

gene=colnames(rt)[1]

clinical=colnames(cli)[1]

outTab=data.frame()

rt1=rt

data=cbind(rt1,gene=rt1[,gene])

data=as.matrix(data[,c(gene,"gene")])

if(nchar(row.names(data)[1])!=nchar(row.names(cli)[1])){

row.names(data)=gsub(".$","",row.names(data))}

data=avereps(data)

sameSample=intersect(row.names(data),row.names(cli))

sameData=data[sameSample,]

sameClinical=cli[sameSample,]

cliExpData=cbind(as.data.frame(sameClinical),sameData)

if(nrow(cliExpData)==0){next}

group=levels(factor(cliExpData$sameClinical))

comp=combn(group,2)

my_comparisons=list()

for(j in 1:ncol(comp)){my_comparisons[[j]]<-comp[,j]}

boxplot=ggboxplot(cliExpData, x="sameClinical", y="gene", color="sameClinical",

xlab=clinical,

ylab=paste(gene,"expression"),

legend.title=clinical,

add = "jitter")+

stat_compare_means(comparisons = my_comparisons)

pdf(file=paste0(clinical,".",".pdf"),width=5.5,height=5)

print(boxplot)

dev.off()

##ggalluvial

install.packages("ggplot2")

install.packages("ggalluvial")

library(ggalluvial)

library(ggplot2)

library(dplyr)

setwd("D:\\autophagy-related ")

rt=read.table("network.txt",sep = "\t",header = T)

cox=read.table("multiCox.txt",sep="\t",header=T,row.names=1)

protectGene=row.names(cox[cox$HR<1,])

riskType=ifelse(rt$lncRNA%in%protectGene,"Protect","Risk")

newData=cbind(rt[,c(1,2)],riskType)

corLodes=to_lodes_form(newData, axes = 1:3, id = "Cohort")

pdf(file="ggalluvial.pdf",width=7,height=6)

mycol <- rep(c("#029149","#6E568C","#E0367A","#D8D155","#223D6C","#D20A13","#431A3D","#91612D","#FFD121","#088247","#11AA4D","#58CDD9","#7A142C","#5D90BA","#64495D","#7CC767"),5)

ggplot(corLodes, aes(x = x, stratum = stratum, alluvium = Cohort,fill = stratum, label = stratum)) +

scale_x_discrete(expand = c(0, 0)) +

geom_flow(width = 1/8,aes.flow = "forward") +

geom_stratum(alpha = .9,width = 1/10) +

scale_fill_manual(values = mycol) +

geom_text(stat = "stratum", size = 2.4,color="black") +

xlab("") + ylab("") + theme_bw() +

theme(axis.line = element_blank(),axis.ticks = element_blank(),axis.text.y = element_blank()) + #去掉坐标轴

theme(panel.grid =element_blank()) +

theme(panel.border = element_blank()) +

ggtitle("") + guides(fill = FALSE)

dev.off()

##Autophagy.GO

install.packages("colorspace")

install.packages("stringi")

install.packages("ggplot2")

if (!requireNamespace("BiocManager", quietly = TRUE))

install.packages("BiocManager")

BiocManager::install("DOSE")

if (!requireNamespace("BiocManager", quietly = TRUE))

install.packages("BiocManager")

BiocManager::install("clusterProfiler")

if (!requireNamespace("BiocManager", quietly = TRUE))

install.packages("BiocManager")

BiocManager::install("enrichplot")

library("clusterProfiler")

library("org.Hs.eg.db")

library("enrichplot")

library("ggplot2")

setwd("D:\\autophagy-related ")

rt=read.table("id.txt",sep="\t",header=T,check.names=F)

rt=rt[is.na(rt[,"entrezID"])==F,]

gene=rt$entrezID

kk <- enrichGO(gene = gene,

OrgDb = org.Hs.eg.db,

pvalueCutoff =0.05,

qvalueCutoff = 0.05,

ont="all",

readable =T)

write.table(kk,file="GO.txt",sep="\t",quote=F,row.names = F)

pdf(file="barplot.pdf",width = 15,height = 8)

barplot(kk, drop = TRUE, showCategory =10,split="ONTOLOGY") + facet_grid(ONTOLOGY~., scale='free')

dev.off()

pdf(file="bubble.pdf",width = 15,height = 8)

dotplot(kk,showCategory = 10,split="ONTOLOGY") + facet_grid(ONTOLOGY~., scale='free')

dev.off()

##Autophagy.GOBubble

install.packages("digest")

install.packages("GOplot")

library(GOplot)

setwd("D:\\autophagy-related ")

ego=read.table("GO.txt", header = T,sep="\t",check.names=F)

go=data.frame(Category = ego$ONTOLOGY,ID = ego$ID,Term = ego$Description, Genes = gsub("/", ", ", ego$geneID), adj_pval = ego$p.adjust)

id.fc <- read.table("id.txt", header = T,sep="\t",check.names=F)

genelist <- data.frame(ID = id.fc$gene, logFC = id.fc$logFC)

row.names(genelist)=genelist[,1]

circ <- circle_dat(go, genelist)

pdf(file="GOBubble.pdf",width = 10,height = 8)

GOBubble(circ, labels = 3,table.legend =F)

dev.off()

pdf(file="GOCircle.pdf",width = 11,height = 6)

GOCircle(circ,rad1=2.5,rad2=3.5,label.size=4,nsub=10)

dev.off()

termNum = 20

geneNum = nrow(genelist)

chord <- chord_dat(circ, genelist[1:geneNum,], go$Term[1:termNum])

pdf(file="GOHeat.pdf",width = 11,height = 5)

GOHeat(chord, nlfc =1, fill.col = c('red', 'white', 'blue'))

dev.off()

##Autophagy.KEGG

install.packages("colorspace")

install.packages("stringi")

install.packages("ggplot2")

if (!requireNamespace("BiocManager", quietly = TRUE))

install.packages("BiocManager")

BiocManager::install("DOSE")

if (!requireNamespace("BiocManager", quietly = TRUE))

install.packages("BiocManager")

BiocManager::install("clusterProfiler")

if (!requireNamespace("BiocManager", quietly = TRUE))

install.packages("BiocManager")

BiocManager::install("enrichplot")

library("clusterProfiler")

library("org.Hs.eg.db")

library("enrichplot")

library("ggplot2")

setwd("D:\\autophagy-related ")

rt=read.table("id.txt",sep="\t",header=T,check.names=F)

rt=rt[is.na(rt[,"entrezID"])==F,]

gene=rt$entrezID

kk <- enrichKEGG(gene = gene, organism = "hsa", pvalueCutoff =0.05, qvalueCutoff =0.05)

write.table(kk,file="KEGGId.txt",sep="\t",quote=F,row.names = F)

pdf(file="barplot.pdf",width = 10,height = 7)

barplot(kk, drop = TRUE, showCategory = 30)

dev.off()

pdf(file="bubble.pdf",width = 10,height = 7)

dotplot(kk, showCategory = 30)

dev.off()

##Autophagy.KEGGcirc

install.packages("digest")

install.packages("GOplot")

library(GOplot)

setwd("D:\\autophagy-related ")

ego=read.table("KEGG.txt", header = T,sep="\t",check.names=F)

go=data.frame(Category = "ALL",ID = ego$ID,Term = ego$Description, Genes = gsub("/", ", ", ego$geneID), adj_pval = ego$p.adjust)

id.fc <- read.table("id.txt", header = T,sep="\t",check.names=F)

genelist <- data.frame(ID = id.fc$gene, logFC = id.fc$logFC)

row.names(genelist)=genelist[,1]

circ <- circle_dat(go, genelist)

pdf(file="KEGGBubble.pdf",width = 10,height = 8)

GOBubble(circ, labels = 3,table.legend =F)

dev.off()

pdf(file="KEGGCircle.pdf",width = 9,height = 6)

GOCircle(circ,rad1=2.5,rad2=3.5,label.size=4,nsub=10)

dev.off()

termNum = 20

geneNum = nrow(genelist)

chord <- chord_dat(circ, genelist[1:geneNum,], go$Term[1:termNum])

pdf(file="KEGGHeat.pdf",width = 9,height = 5)

GOHeat(chord, nlfc =1, fill.col = c('red', 'white', 'blue'))

dev.off()

##riskSurvival

install.packages("survival")

library(survival)

setwd("D:\\autophagy-related ")

data=read.table("n12.txt",header=T,sep="\t",check.names=F)

diff=survdiff(Surv(futime, fustat) ~risk,data = data)

pValue=1-pchisq(diff$chisq,df=1)

pValue=signif(pValue,4)

pValue=format(pValue, scientific = TRUE)

fit <- survfit(Surv(futime, fustat) ~ risk, data = data)

pdf(file="n12.pdf",width=5.5,height=5)

plot(fit,

lwd=2,

col=c("red","blue"),

xlab="Time (year)",

ylab="Survival rate",

main=paste("Survival curve (p=", pValue ,")",sep=""),

mark.time=T)

legend("topright",

c("High risk", "Low risk"),

lwd=2,

col=c("red","blue"))

dev.off()

##riskSurvival

install.packages("survival")

library(survival)

setwd("D:\\autophagy-related ")

data=read.table("risk.txt",header=T,sep="\t",check.names=F)

diff=survdiff(Surv(futime, fustat) ~risk,data = data)

pValue=1-pchisq(diff$chisq,df=1)

pValue=signif(pValue,4)

pValue=format(pValue, scientific = TRUE)

fit <- survfit(Surv(futime, fustat) ~ risk, data = data)

pdf(file="survival.pdf",width=5.5,height=5)

plot(fit,

lwd=2,

col=c("red","blue"),

xlab="Time (year)",

ylab="Survival rate",

main=paste("Survival curve (p=", pValue ,")",sep=""),

mark.time=T)

legend("topright",

c("High risk", "Low risk"),

lwd=2,

col=c("red","blue"))

dev.off()

summary(fit)

##riskplot

install.packages("pheatmap")

library(pheatmap)

setwd("D:\\autophagy-related ")

rt=read.table("risk.txt",sep="\t",header=T,row.names=1,check.names=F)

rt=rt[order(rt$riskScore),]

riskClass=rt[,"risk"]

lowLength=length(riskClass[riskClass=="low"])

highLength=length(riskClass[riskClass=="high"])

line=rt[,"riskScore"]

line[line>10]=10

pdf(file="riskScore.pdf",width = 10,height = 3.5)

plot(line,

type="p",

pch=20,

xlab="Patients (increasing risk socre)",

ylab="Risk score",

col=c(rep("green",lowLength),

rep("red",highLength)))

abline(h=median(rt$riskScore),v=lowLength,lty=2)

legend("topleft", c("High risk", "low Risk"),bty="n",pch=19,col=c("red","green"),cex=1.2)

dev.off()

color=as.vector(rt$fustat)

color[color==1]="red"

color[color==0]="green"

pdf(file="survStat.pdf",width = 10,height = 3.5)

plot(rt$futime,

pch=19,

xlab="Patients (increasing risk socre)",

ylab="Survival time (years)",

col=color)

legend("topleft", c("Dead", "Alive"),bty="n",pch=19,col=c("red","green"),cex=1.2)

abline(v=lowLength,lty=2)

dev.off()

rt1=log2(rt[c(3:(ncol(rt)-2))]+0.01)

rt1=t(rt1)

annotation=data.frame(type=rt[,ncol(rt)])

rownames(annotation)=rownames(rt)

pdf(file="heatmap.pdf",width = 10,height = 3)

pheatmap(rt1,

annotation=annotation,

cluster_cols = FALSE,

fontsize_row=11,

show_colnames = F,

fontsize_col=3,

color = colorRampPalette(c("green", "black", "red"))(50) )

dev.off()

##risk-Forest

install.packages('survival')

library(survival)

setwd("D:\\autophagy-related ")

rt=read.table("indepInput.txt",header=T,sep="\t",check.names=F,row.names=1)

uniTab=data.frame()

for(i in colnames(rt[,3:ncol(rt)])){

cox <- coxph(Surv(futime, fustat) ~ rt[,i], data = rt)

coxSummary = summary(cox)

uniTab=rbind(uniTab,

cbind(id=i,

B=coxSummary$coefficients[,"coef"],

SE=coxSummary$coefficients[,"se(coef)"],

z=coxSummary$coefficients[,"z"],

HR=coxSummary$conf.int[,"exp(coef)"],

HR.95L=coxSummary$conf.int[,"lower .95"],

HR.95H=coxSummary$conf.int[,"upper .95"],

pvalue=coxSummary$coefficients[,"Pr(>|z|)"])

)

}

write.table(uniTab,file="uniCox.txt",sep="\t",row.names=F,quote=F)

multiCox=coxph(Surv(futime, fustat) ~ ., data = rt)

multiCoxSum=summary(multiCox)

multiTab=data.frame()

multiTab=cbind(

B=multiCoxSum$coefficients[,"coef"],

SE=multiCoxSum$coefficients[,"se(coef)"],

z=multiCoxSum$coefficients[,"z"],

HR=multiCoxSum$conf.int[,"exp(coef)"],

HR.95L=multiCoxSum$conf.int[,"lower .95"],

HR.95H=multiCoxSum$conf.int[,"upper .95"],

pvalue=multiCoxSum$coefficients[,"Pr(>|z|)"])

multiTab=cbind(id=row.names(multiTab),multiTab)

write.table(multiTab,file="multiCox.txt",sep="\t",row.names=F,quote=F)

bioForest=function(coxFile=null,forestCol=null,forestFile=null){

rt <- read.table(coxFile,header=T,sep="\t",row.names=1,check.names=F)

gene <- rownames(rt)

hr <- sprintf("%.3f",rt$"HR")

hrLow <- sprintf("%.3f",rt$"HR.95L")

hrHigh <- sprintf("%.3f",rt$"HR.95H")

Hazard.ratio <- paste0(hr,"(",hrLow,"-",hrHigh,")")

pVal <- ifelse(rt$pvalue<0.001, "<0.001", sprintf("%.3f", rt$pvalue))

pdf(file=forestFile, width = 6,height = 4.2)

n <- nrow(rt)

nRow <- n+1

ylim <- c(1,nRow)

layout(matrix(c(1,2),nc=2),width=c(3,2.5))

xlim = c(0,3)

par(mar=c(4,2.5,2,1))

plot(1,xlim=xlim,ylim=ylim,type="n",axes=F,xlab="",ylab="")

text.cex=0.8

text(0,n:1,gene,adj=0,cex=text.cex)

text(1.5-0.5*0.2,n:1,pVal,adj=1,cex=text.cex);text(1.5-0.5*0.2,n+1,'pvalue',cex=text.cex,font=2,adj=1)

text(3,n:1,Hazard.ratio,adj=1,cex=text.cex);text(3,n+1,'Hazard ratio',cex=text.cex,font=2,adj=1,)

par(mar=c(4,1,2,1),mgp=c(2,0.5,0))

xlim = c(0,max(as.numeric(hrLow),as.numeric(hrHigh)))

plot(1,xlim=xlim,ylim=ylim,type="n",axes=F,ylab="",xaxs="i",xlab="Hazard ratio")

arrows(as.numeric(hrLow),n:1,as.numeric(hrHigh),n:1,angle=90,code=3,length=0.05,col="darkblue",lwd=2.5)

abline(v=1,col="black",lty=2,lwd=2)

boxcolor = ifelse(as.numeric(hr) > 1, forestCol, forestCol)

points(as.numeric(hr), n:1, pch = 15, col = boxcolor, cex=1.3)

axis(1)

dev.off()

}

bioForest(coxFile="uniCox.txt", forestCol="green", forestFile="uniForest.pdf")

bioForest(coxFile="multiCox.txt", forestCol="red", forestFile="multiForest.pdf")

##ROC curve

install.packages("survivalROC")

library(survivalROC)

setwd("D:\\autophagy-related ")

rt=read.table("indepInput.txt",header=T,sep="\t",check.names=F,row.names=1)

rocCol=rainbow(ncol(rt)-2)

aucText=c()

pdf(file="multiROC.pdf",width=6,height=6)

par(oma=c(0.5,1,0,1),font.lab=1.5,font.axis=1.5)

roc=survivalROC(Stime=rt$futime, status=rt$fustat, marker = rt$riskScore, predict.time =1, method="KM")

plot(roc$FP, roc$TP, type="l", xlim=c(0,1), ylim=c(0,1),col=rocCol[1],

xlab="False positive rate", ylab="True positive rate",

lwd = 2, cex.main=1.3, cex.lab=1.2, cex.axis=1.2, font=1.2)

aucText=c(aucText,paste0("risk score"," (AUC=",sprintf("%.3f",roc$AUC),")"))

abline(0,1)

j=1

for(i in colnames(rt[,3:(ncol(rt)-1)])){

roc=survivalROC(Stime=rt$futime, status=rt$fustat, marker = rt[,i], predict.time =1, method="KM")

j=j+1

aucText=c(aucText,paste0(i," (AUC=",sprintf("%.3f",roc$AUC),")"))

lines(roc$FP, roc$TP, type="l", xlim=c(0,1), ylim=c(0,1),col=rocCol[j],lwd = 2)

}

legend("bottomright", aucText,lwd=2,bty="n",col=rocCol)

dev.off()

##nomogram

setwd("D:\\autophagy-related ")

library(rms)

library(foreign)

library(survival)

tcga<-read.table("clinical.txt",header=T,sep="\t")

names(tcga)

#tcga$age<-factor(tcga$age,labels=c("<50","50-59","60-69",">=70"))

tcga$gender<-factor(tcga$gender,levels=c(0,1),labels=c("FEMALE","MALE"))

tcga$grade<-factor(tcga$grade,labels=c("1","2","3",'4'))

#tcga$smoking<-factor(tcga$smoking,labels=c("1","2","3","4","5"))

#tcga$radiation<-factor(tcga$radiation,labels=c("YES","NO"))

#tcga$pharmaceutical<-factor(tcga$pharmaceutical,labels=c("YES","NO"))

tcga$stage<-factor(tcga$stage,levels=c(1,2,3,4),labels=c("1","2","3",'4'))

tcga$T<-factor(tcga$T,labels=c("1","2","3",'4'))

tcga$N<-factor(tcga$N,labels=c("N0","N1","N2"))

#tcga$surgery<-factor(tcga$surgery,labels=c("YES","NO"))

ddist <- datadist(tcga)

options(datadist='ddist')

cox <- cph(Surv(futime,fustat) ~age + gender + grade + stage + T + N + riskScore,surv=T,x=T, y=T,data=tcga)

surv <- Survival(cox)

surv <- Survival(cox)

sur_3_year<-function(x)surv(1*365*3,lp=x)

sur_1_year<-function(x)surv(1*365*1,lp=x)

nom_sur <- nomogram(cox,fun=list(sur_1_year,sur_3_year),lp= F,funlabel=c('1-Year Survival','3-Year survival'),maxscale=100,fun.at=c('0.9','0.7','0.5','0.3',"0.1"))

pdf("nom.pdf",15,10)

plot(nom_sur,xfrac=0.25)

dev.off()

nom_sur

##Calibration

setwd("D:\\autophagy-related ")

library(rms)

library(foreign)

library(survival)

tcga<-read.table("clinical.txt",header=T,sep="\t")

tcga$age<-factor(tcga$age,labels=c("<50","50-59","60-69",">=70"))

tcga$sex<-factor(tcga$sex,labels=c("FEMALE","MALE"))

tcga$race<-factor(tcga$race,labels=c("WHITE","BLACK OR AFRICAN AMERICAN","ASIAN","AMERICAN INDIAN OR ALASKA NATIVE"))

tcga$smoking<-factor(tcga$smoking,labels=c("1","2","3","4","5"))

tcga$radiation<-factor(tcga$radiation,labels=c("YES","NO"))

tcga$pharmaceutical<-factor(tcga$pharmaceutical,labels=c("YES","NO"))

tcga$stage_T<-factor(tcga$stage_T,labels=c("T1","T1a","T1b","T1c","T2","T2a","T2b","T3","T4","TX"))

tcga$stage_M<-factor(tcga$stage_M,labels=c("M0","M1","M1a","M1b","MX"))

tcga$stage_N<-factor(tcga$stage_N,labels=c("N0","N1","N2","N3","NX"))

tcga$surgery<-factor(tcga$surgery,labels=c("YES","NO"))

cox1 <- cph(Surv(futime,fustat)~age + gender + grade + stage + T + N + riskScore,surv=T,x=T, y=T,time.inc = 1*365*3,data=tcga)

cal <- calibrate(cox1, cmethod="KM", method="boot", u=1*365*3, m= 30, B=1000)

pdf("calibrate3.pdf",12,8)

par(mar = c(10,5,3,2),cex = 1.0)

plot(cal,lwd=3,lty=2,errbar.col="black",xlim = c(0,1),ylim = c(0,1),xlab ="Nomogram Predicted Survival",ylab="Actual Survival",col="blue")

lines(cal,c('mean.predicted','KM'),type = 'a',lwd = 3,col ="black" ,pch = 16)

mtext('')

box(lwd = 1)

abline(0,1,lty = 3,lwd = 3,col = "black")

dev.off()
